# Polymorphism in the structure of *N*-(5-methyl­thia­zol-2-yl)-4-oxo-4*H*-chromene-3-carboxamide

**DOI:** 10.1107/S2056989017009902

**Published:** 2017-07-13

**Authors:** Ligia R. Gomes, John Nicolson Low, Fernando Cagide, Fernanda Borges

**Affiliations:** a FP-ENAS-Faculdade de Ciências de Saúde, Escola Superior de Saúde da UFP, Universidade Fernando Pessoa, Rua Carlos da Maia, 296, P-4200-150 Porto, Portugal; b Department of Chemistry, University of Aberdeen, Meston Walk, Old Aberdeen, AB24 3UE, Scotland; c CIQ/Departamento de Quιmica e Bioquιmica, Faculdade de Ciências, Universidade do Porto, 4169-007 Porto, Portugal

**Keywords:** crystal structure, drug design, chromones, conformation, supra­molecular structure

## Abstract

Chromone derivatives have been extensively studied recently because of to their promising biological activities. The new title chromone–thia­zole hybrid presented here, C_14_H_10_N_2_O_3_S, is a candidate as a selective ligand for adenosine receptors. The compound has been synthesized and characterized by the usual spectroscopic means (NMR and EM/IE) and its structure elucidated by X-ray crystallography, which revealed the presence of packing polymorphism. The two polymorphs (one with space group *P*2_1_/*n* and one with *P*2_1_/*c*) show slightly different conformations and the major change induced by crystallization regards the intra­molecular contacts defining the supra­molecular structure. Those differences been highlighted by Hirshfeld surface analysis mapped over *d*_norm_ and ESP.

## Abstract 2

The new chromone–thia­zole hybrid presented here is a candidate as a selective ligand for adenosine receptors. Its structure shows packing polymorphism: the two polymorphs (one with space group *P*2_1_/*n* and one with *P*2_1_/*c*) show slightly different conformations and the major change induced by crystallization regards the intra­molecular contacts defining the supra­molecular structure.

## Chemical context   

Chromones are 4*H*-benzo­pyran-4-one heterocycles and they have been studied thoroughly because of their inter­esting biological activities (Gaspar *et al.*, 2012*a* ▸,*b* ▸; 2014 ▸) Thia­zole-based compounds have been used in therapeutics as anti­microbial, anti­viral and anti­fungal agents for a long time (Souza, 2005 ▸; Siddiqui *et al.*, 2009 ▸ *)* but, in the past decades, they have been identified as potent and selective ligands for the adenosine receptor (Sharma *et al.* 2009 ▸; Jung *et al.*, 2004 ▸ *)*. In a continuation of our project related to the synthesis of pharmacologically useful heterocycles, the title compound has been designed as a potential ligand for human adenosine receptors.
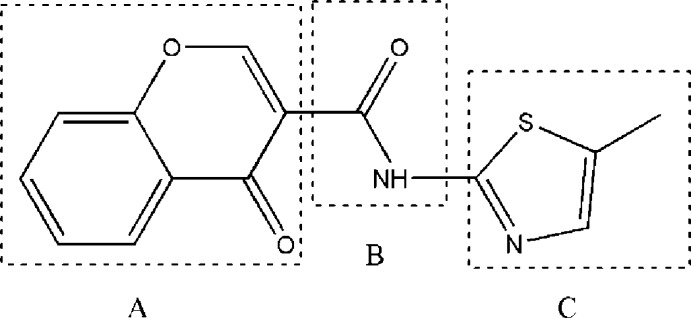


This work reports the synthesis and structural characterization of this chromone–thia­zole hybrid, *N*-(5-methyl­thia­zol-2-yl)-4-oxo-4*H*-chromene-3-carboxamide, **1**, that was synthesized following a method previously described by Cagide *et al.* (2015 ▸). The chromone ring (*A*) is connected to the thia­zole ring (*C*) though a carboxamide spacer (*B*). The compound crystallizes with two different morphologies and the structural analysis revealed the presence of packing polymorphism. Since this compound will be assayed in tests to evaluate its biological activity, the establishment of the polymorphic structures is of the utmost importance.

## Structural commentary   

**Figure 1:**
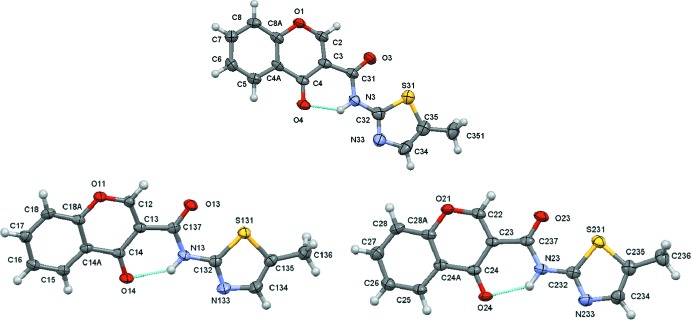
A view of the asymmetric unit of **1_** ***P*** **2_1_/** ***n*** with the atom-numbering scheme (top). A view of the asymmetric unit of **1_** ***P*** **2_1_/** ***c*** with mol#1 (left) and mol#2 (right) with the atom-numbering scheme (bottom). Displacement ellipsoids are drawn at the 70% probability level.

The mol­ecular structures of the polymorphs are shown in Fig. 1 ▸. This compound presents packing polymorphism and crystallizes in monoclinic space groups *P*2_1_/*n* and *P*2_1_/*c*, the latter with two mol­ecules in the asymmetric unit (identified as mol#1 and mol#2). In **1_** ***P*** **2_1_/** ***c***, mol#1 fits into mol#2 with values of quaternion fit weighted of 0.093 Å (unit-weight r.m.s. fit of 0.086 Å for 20 atoms).

The conformation around the amide rotamer for chromone carboxamides can be either -*anti* or -*syn*. The former appears to be more probable since it lowers the steric hindrance between the two aromatic rings as compared to the -*syn* rotamer. Structural characterizations made previously in other 4*H*-chromene-3-carboxamides (Gomes *et al.*, 2015*a* ▸,*b* ▸) show that, when the amide oxygen atom (O3/O13/O23) is *trans-*related to the pyran oxygen atom of the chromone (O1/O11/O21) the -*anti* conformation predominates since it permits the establishment of a short intra­molecular N—H⋯O(carbon­yl) hydrogen bond (geometric parameters for the intra­molecular H bond are given in Tables 1 ▸ and 2 ▸), which generates an *S*(6) ring.

**Figure 2:**
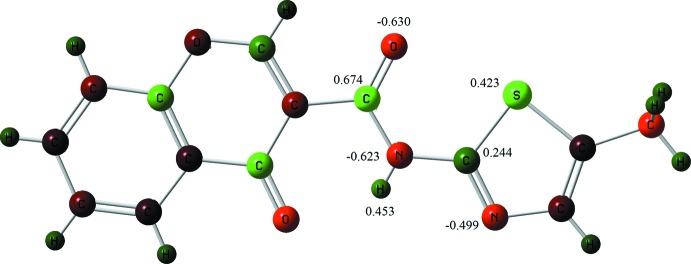
Natural atomic charges from population analysis (NBO), at the B3LYP/6–311+G(*d*) level of theory for the **1_** ***P*** **2_1_/** ***n*** at crystal geometric conformation. The charge distributions are presented within a relative charge range of −1.000 (green) to +1.000 (light red).

The S atom of the thia­zole ring is in a -*cis* position with respect to the carbonyl O3/O23/O13 atom of the amide in both polymorphs. This feature has also been observed for similar compounds (Cagide *et al.*, 2015 ▸). Gas-phase *ab initio* geometry optimization and natural atomic charges obtained by population analysis [using natural bond orbital (NBO) analysis] revealed that negative charges are located at the two nitro­gen atoms and at the three oxygen atoms, while the positive charges reside at the amide carbon atom as well as at the sulfur atom of the thia­zole ring, suggesting that a further stabilization may arise when the S atom is pointing to the carboxyl oxygen atom of the amide. This was also confirmed here by similar calculations: the results obtained for atomic charges by NBO analysis, performed after single-point energy calculation, are in Fig. 2 ▸. In addition, the calculation of energies and charges of several conformers, obtained by rotation of the thia­zole ring (*C*) around the amide spacer (*B*) were made, showing that the lowest energy is obtained when the sulfur atom is around 0°. Details are provided in the Supporting information.

Relevant data for the discussion of mol­ecular geometry and conformation of the polymorphs is presented in Table 3 ▸; θ_A–C_ refers to the dihedral angle between the mean planes of the chromone and thia­zole rings, θ_A–B_ to the dihedral angle between the best plane of the chromone and the plane defined by atoms OCN of the amide moiety, whereas θ_B–C_ refers to the dihedral angle between this plane and the best plane of the thia­zole ring. Since the heteroaromatic rings are practically planar, the dihedral angle θ_A–C_ qu­anti­fies the degree of bend and/or twist between them and can be used for evaluation of the distortion of the mol­ecule from planarity when one of the dihedrals, θ_A–B_ or θ_B–C_, is relatively small. As seen, **1_** ***P*** **2_1_/** ***n*** and **1_** ***P*** **2_1_/** ***c***_mol#2 are practically planar while **1_P21/c**_mol#1 presents a slightly higher θ_A–C_ angle due to the rotation of the chromone ring with respect to the amide plane.

## Supra­molecular features   

***Mol­ecular structure and conformation***

**Figure 3:**
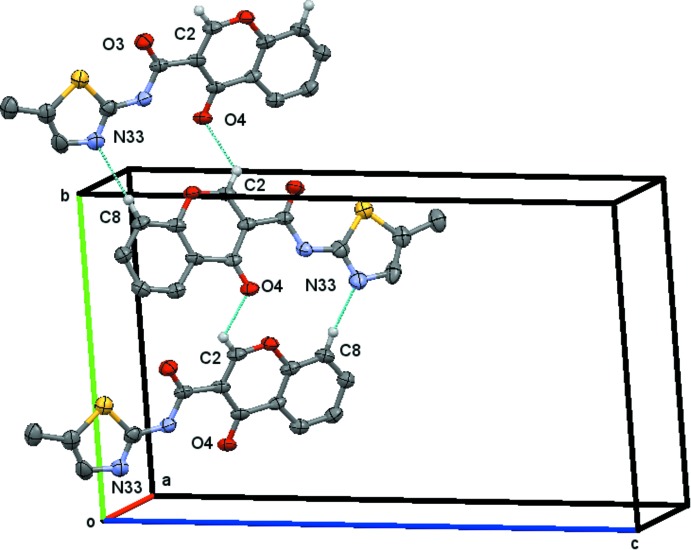
The chain of 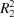
(13) rings running parallel to the *b* axis generated by the twofold screw axis at *x* = 1/4 and *y* = 1/4 as depicted for **1_** ***P*** **2_1_/** ***n***. H atoms not participating in hydrogen bonding have been omitted for the sake of clarity.

In **1_** ***P*** **2_1_/** ***n*** the mol­ecules are linked by the C2—H2⋯O4^i^ and C8—H8⋯N33^i^ weak hydrogen bonds, Table 1 ▸, which form a chain of 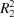 (13) rings runing parallel to the *b*-axis direction generated by the twofold screw axis at *x* = 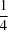 and *y* = 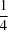, as depicted in Fig. 3 ▸.

**Figure 4:**
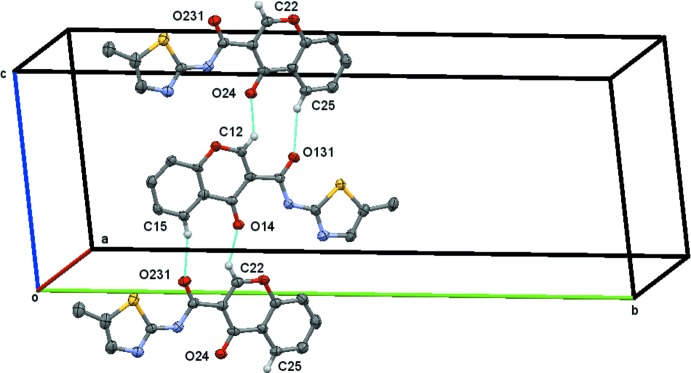
The mol­ecules in **1_** ***P*** **2_1_/** ***c*** linked by alternating weakly hydrogen-bonded 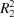
(10) rings that lead the mol­ecules to form a chain of rings running parallel to [101]. H atoms not participating in hydrogen bonding have been omitted for the sake of clarity.

The mol­ecules in **1_** ***P*** **2_1_/** ***c*** are linked by alternating weakly hydrogen-bonded 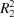
(10) rings formed by the hydrogen bonds C12—H12⋯O24^ii^ and C25—H25⋯O13^iv^ in one case and C22—H22⋯O14^ii^ and C15—H15⋯O23^ii^ in the other, Table 2 ▸. These link the mol­ecules to form a chain of rings running parallel to [101], Fig. 4 ▸. Details of the π–π stacking are given in Table 4 ▸. In **1_** ***P*** **2_1_/** ***n*** the mol­ecules form a π–π stack that extends along the *a* axis*.* In **1_** ***P*** **2_1_/** ***c***, the two mol­ecules in the asymmetric unit form a π–π stacked dimer (which guided the choice of asymmetric unit). In both compounds, any possible C—H⋯π contacts involve methyl hydrogen atoms with H⋯π distances in excess of 2.8 Å.

***Hirshfeld surfaces***

**Figure 5:**
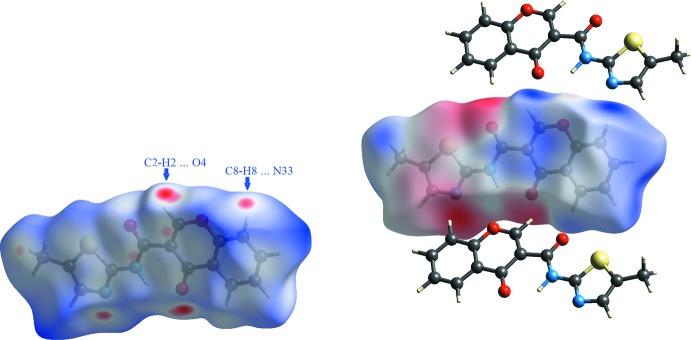
Views of the Hirshfeld surface mapped over *d*_norm_ (left) and mapped over the electrostatic potential (right) for **1_** ***P*** **2_1_/** ***n***. The highlighted red spots on the top face of the surfaces indicate contact points with the atoms participating in the C—H⋯O/N inter­molecular inter­actions whereas those on the middle of the surface corresponds to C⋯C contacts as a consequence of the π–π stacking. The electrostatic potential surface (ranging from −0.077 to 0.066) shows the complementary electronegative (red) and electropositive areas (blue) with mol­ecules of the first shell. They depict the importance of the H2 and H8 atoms of the chromone ring that are located in the most electropositive area and their connection to O4 and N33. The methyl group presents also an electropositive region that complements with the thio­zole environment near the sulfur atom.

The Hirshfeld surfaces and two-dimensional fingerprint (FP) plots (Rohl *et al.*, 2008 ▸ *)* provide complementary information concerning the inter­molecular inter­actions discussed above. They were generated using *Crystal Explorer 3.1* (Wolff *et al.*, 2012 ▸). The Hirshfeld surfaces, mapped over *d*_norm_ (all scaled between −0.250 to 1.200) and the respective FP plots are depicted in Figs. 5 ▸ and 6 ▸ for **1_** ***P*** **2_1_/** ***n*** and in Figs. 7 ▸ and 8 ▸ for **1_** ***P*** **2_1_/** ***c***; mol_**#1** and mol_**#2**. Also in Figs. 5 ▸ and 9 ▸, the Hirshfeld surfaces mapped over the electrostatic potential (ESP) are depicted for both polymorphs. The contributions from various contacts, listed in Table 5 ▸, were selected by the partial analysis of those FP plots. Taking out the H⋯H contacts on the surface that are inherent to organic mol­ecules, the most significant contacts can be divided in three groups: (i) the H⋯O/N contacts that correspond to some relevant C—H⋯O, C—H⋯N inter­molecular inter­actions; (ii) the H⋯C/C⋯H contacts and (iii) C⋯C contacts that are related to π–π stacking. The structure has two carboxyl groups and a nitro­gen atom of the thia­zole that can act as acceptors for hydrogen bonding and a N—H (amide) that can act as donor. In spite of that, the N—H amide does not have a relevant role in the definition of the supra­molecular structure but it is compromised in the inter­molecular hydrogen bond.

***P*** **2_1_/** ***n*** **polymorph**

**Figure 6:**
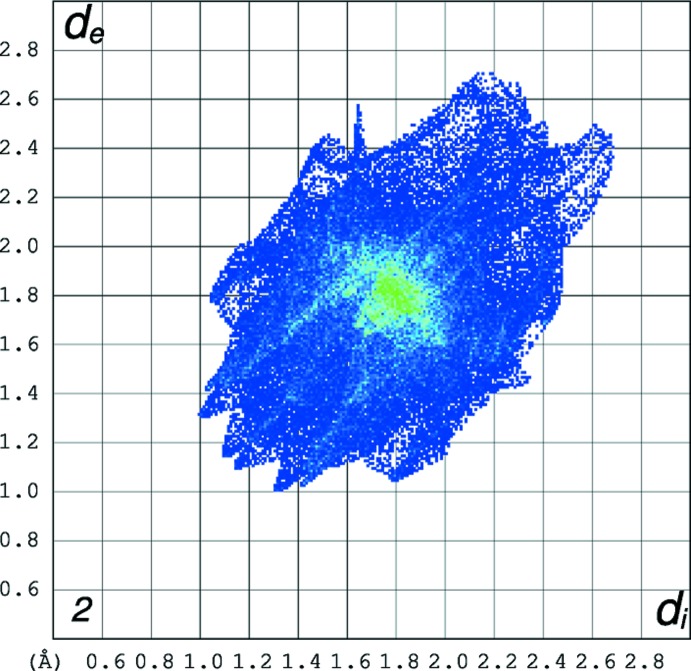
The FP plot for **1_** ***P*** **2_1_/** ***n***; the light-blue area in the middle of the FP plot is due to C⋯C contacts (7.1% of the area). The FP plot shows three sets of spikes pointing to southwest due to weak C⋯H contacts: the outer sharper ones are due to the H.·N contacts that involves the N(thia­zole)⋯H8—C8(chromone) inter­action followed by the spikes corresponding to O⋯H contacts that englobe the O4⋯H2_C2 contacts and the inner one is due to close S⋯H contacts

As seen in Fig. 3 ▸, in **1_** ***P*** **2_1_/** ***n*** the oxygen atom O4 acts as acceptor for the hydrogen atom H2 of the chromone and the nitro­gen atom N33 of the thia­zole ring acts as acceptor for the H8 hydrogen atom of the chromone ring. Thus, the Hirshfeld surface of **1_** ***P*** **2_1_/** ***n*** (mapped with *d*_norm_) shows two sets of complementary red spots in the lateral faces of the surface as highlighted in Fig. 5 ▸, left. The small red-spot areas facing the chromone plane are due to C⋯C contacts (that assume 7.1% of the contact area) and they correspond to the light-blue area in the middle of the FP plot, Fig. 6 ▸. The geometric parameters for these contacts are listed in Tables 3 ▸ and 5 ▸. The weak C⋯H contacts correspond to 15.2% of the surface area. The FP plot shows three sets of spikes pointing to southwest: the outer ones are due to the H⋯N contacts that involves the N(thia­zole)⋯H8—C8(chromone) followed by the spikes corresponding to O⋯H contacts that englobes the O4⋯H2—C2 contacts and the inner one is due to close S⋯H contacts where the closest one is with the H atoms of the methyl group. A small red spot pointing to this group appears in the Hirshfeld surface, Fig. 5 ▸, left.

In Fig. 5 ▸ right, the mapping of the mol­ecular electrostatic potential (ESP) in the context of crystal packing is shown. As the Hirshfeld surface partitions of the crystal space give non-overlapping volumes associated with each mol­ecule these surfaces give a kind of ‘electrostatic complementarity’. The mol­ecular ESP for ***P*** **2_1_/** ***n*** reveals red regions of strongly negative electrostatic potential surrounding the two carbonyl regions and the azo region of the thia­zole fragment. The blue region is electropositive and it is predominantly located in the chromone area near the H2 and H8 hydrogen atoms as well as in the methyl group of the thia­zole. The remainder of the Hirshfeld surface is close to neutrality as seen by the grey regions. It is inter­esting to note that the mapped areas with electronegative potential corresponding to the areas covered by the atoms exhibiting negative natural atomic charges as computed by NBO (as seen in Fig. 2 ▸) with exception for the thia­zole sulfur atom, which assumes a positive value by adiabatic gas-phase calculations, but gives a slightly negative electrostatic potential area at the Hirsfeld surface. The calculated partial charges show how the mol­ecule would inter­act with an approaching proton and the mol­ecular electrostatic potential is the potential energy that a proton would acquire at the surface, that is depending on the distance to the core nucleus of the mol­ecule, suggesting that, in the crystal the sulfur surroundings experiences a deeper change in the eletrostatic potential gradient than that occurring in the remaining mol­ecule, as compared with that of the adiabatic conditions.

Fig. 5 ▸ also highlights the electrostatic complementarity of the C—H⋯O/N contacts between the mol­ecules. The electropositive (blue) patch above the chromone ring is in contact with the electronegative (red) regions around the carbonyl oxygen atom of the chromone O4 and the nitro­gen atom of the thia­zole ring N33 while the carbonyl oxygen atom of the amide O3 is pointing to the H5 hydrogen atom of the chromone ring. The electronegativity of this oxygen is lower than the electronegativity of the O4 of the chromone or the nitro­gen atom of the thia­zole N33. Thus the first shell mol­ecular pairs are clearly associated with hydrogen bonds around the chromone ring periphery.

***P*** **2_1_/** ***c*** **(mol#1 and #2) polymorph**

**Figure 7:**
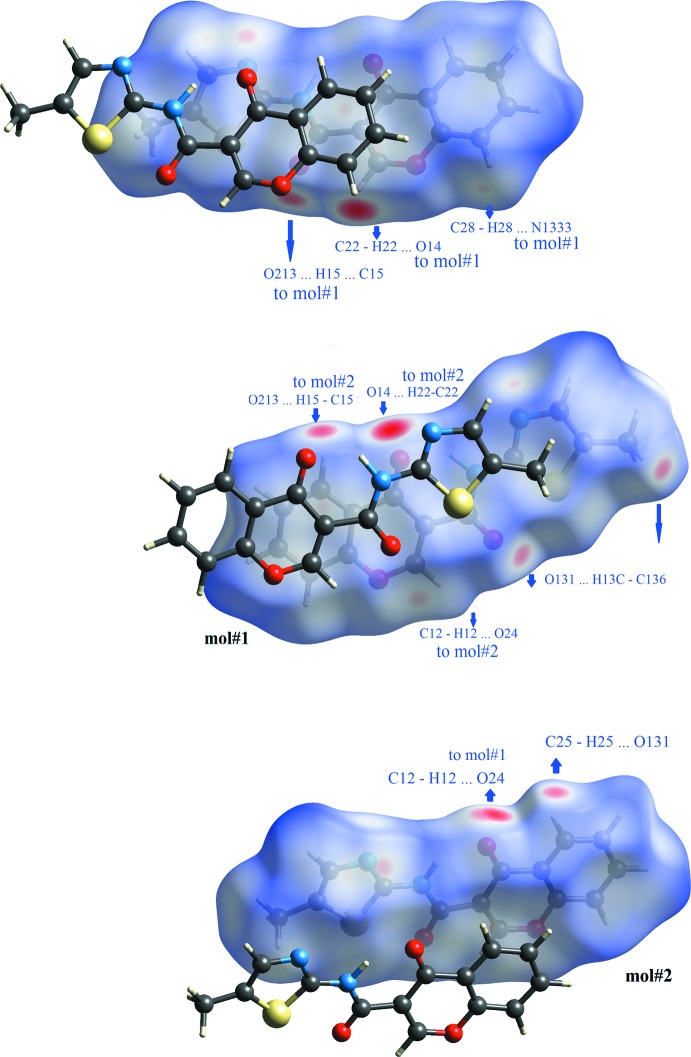
Views of the Hirshfeld surface mapped over *d*_norm_ for **1_** ***P*** **2_1_/** ***c***. The highlighted red spots on the top face of the surfaces indicate contact points with the atoms participating in the C—H⋯O/N inter­molecular inter­actions. The red spot identified as a C12—H12⋯O24 contact in mol#1 is located on the hidden face of the surface.

The Hirshfeld surfaces printed over *d*_norm_ for each mol­ecule are shown in Fig. 7 ▸. Those surfaces show complementary red spots with each other; since mol#1 is linked to mol#2 and *vice versa*, they map pairs of dimers that connect the mol­ecules in chains. Here, the hydrogen bonds that contribute to the linking of the mol#1 with mol#2 are the following: (i) the oxo oxygen atom of the chromone of mol#1 acts as acceptor for the H2 hydrogen atom of the chromone of mol#2 (O14⋯H22—C22) and *vice versa* (O24⋯H12—C12); (ii) the carboxyl oxygen atom of the amide in mol#1 links the hydrogen atom H5 of the chromone ring in mol#2 (O13⋯H25—C25) and *vice versa* (O23⋯H15—C15); (iii) the nitro­gen atom of the thia­zole in mol#1 acts as acceptor for H8 hydrogen atom of mol#2 (N133⋯H28—C28). The O13⋯H25—C25/ O23⋯H15—C15 bond pair was not present in **1_** ***P*** **2_1_/** ***n*** polymorph while the remaining two were also observed. There is another pair of blue spots in the Hirshfeld surface of mol#1that are complementary in shape and they refer to the O13⋯H13*C*—C136 contact.

**Figure 8:**
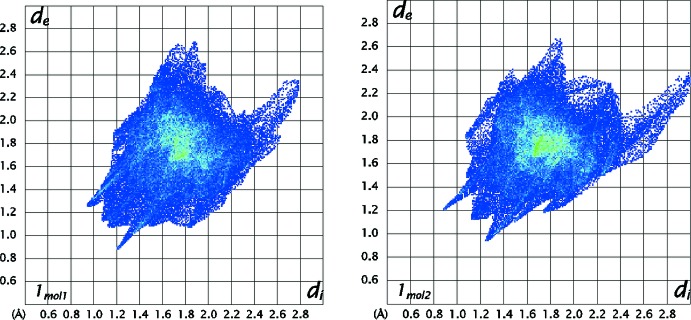
The FP plot for **1_** ***P*** **2_1_/** ***c***, mol#1 on left and mol#2 on right; The light-blue area in the middle of the FP plot at *d*_e_/*d*_i_ ∼1.8 Å shows a higher frequency of the pixels that are due to C⋯C contacts (5.2% of the area for each mol­ecule). The spikes pointing to southwest are due to weak O⋯H contacts. The asymmetric tails that both present are corresponding to N⋯H contacts in mol#1. Their asymmetry is due to the fact that they connect two mol­ecules that are not related by crystallographic symmetry.

The FP plots for polymorph **1_** ***P*** **2_1_/** ***c*** (mol#1 and #2) are shown in Fig. 8 ▸. The FP plots highlight the differences in distribution of close contacts between mol#1 and mol#2. The asymmetric tails that are both present correspond to N⋯H contacts in mol#1 and the sharp spikes are due to the O⋯H contacts. Their asymmetry is due to the fact that they connect two mol­ecules that are not related by crystallographic symmetry. The sharper line in mol#1 FP that ends at about (1.2;0.9) corresponds to O⋯H contacts that mol#1 makes with mol#2. Those contacts relate to the ones given by the sharper line that ends at about (0.9; 1.2) in the FP of mol#2. It is noticeable the differences in sharpness of the O⋯H spikes presented in the FP plots **1_** ***P*** **2_1_/** ***c*** when compared with the FP plot of the polymorph **1_** ***P*** **2_1_/** ***n*** showing that in **1_** ***P*** **2_1_/** ***c*** the O⋯H contacts are more directional and shorter. Those plots also reflect the differences regarding the close contacts between mol­ecules: the light blue/green area in the middle of the FP plot in **1_** ***P*** **2_1_/** ***n*** is less spread out and more intense that the area presented in the FP plot of **1_** ***P*** **2_1_/** ***c*** suggesting that the C⋯C close contacts are more relevant in first polymorph.

**Figure 9:**
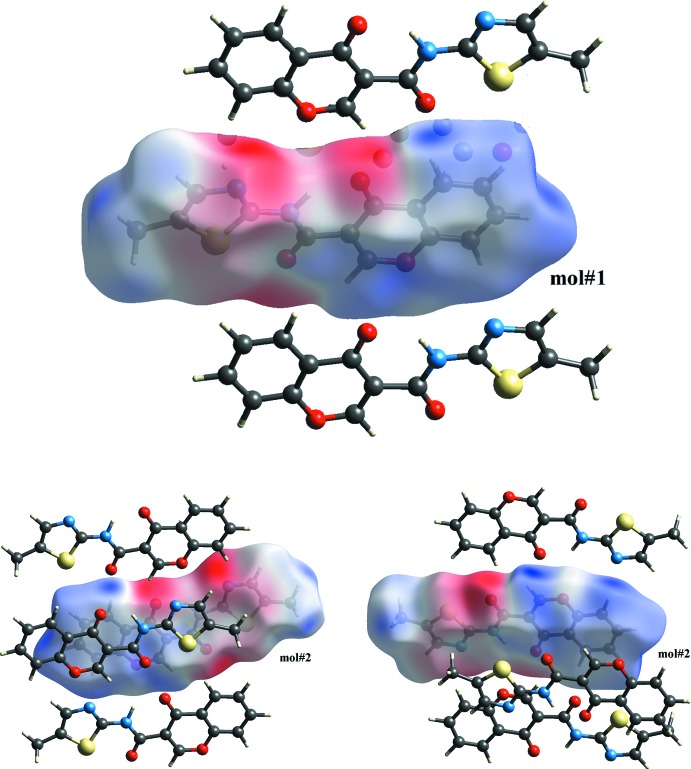
The electrostatic potential surfaces for **1_** ***P*** **2_1_/** ***c***, mol#1 and mol#2. The surfaces show the complementary electronegative (red) and electropositive areas (blue) with mol­ecules of the first shell (ranging from −0.077 to 0.066). The ESP is electronegative in the vicinity of *oxo* oxygen atoms and of the nitro­gen atom of the thia­zole ring while it is electropositive in the areas that surrounds the H2, H5 and H8 hydrogen atoms of the chromone ring.

Fig. 9 ▸ depicts the Hirshfeld surfaces mapped over the electrostatic potential and once again the complementary electrostatic nature of the contacts are clear from the figure. The ESP is electronegative in the vicinity of oxo oxygen atoms and of the nitro­gen atom of the thia­zole ring while it is electropositive in the areas that surrounds the H2, H5 and H8 hydrogen atoms of the chromone ring.

## Synthesis and crystallization   

Chromone-3-carb­oxy­lic acid, phospho­rus(V) oxychloride, di­methyl­formamide (DMF) and 5-methyl­thia­zol-2-amine were purchased from Sigma–Aldrich Química S·A. (Sintra, Portugal). All other reagents and solvents were pro analysis grade and used without additional purification. Thin-layer chromatography (TLC) was carried out on precoated silica gel 60 F254 (Merck) with layer thickness of 0.2 mm and ethyl acetate/petroleum ether as the mobile phase. The spots were visualized under UV detection (254 and 366 nm) and iodine vapour. Flash chromatography was performed using silica gel 60 0.2–0.5 or 0.040–0.063 mm (Carlo Erba Reagents).

***Synthesis of N-(5-methyl­thia­zol-2-yl)-4-oxo-4H-chromene-3-carboxamide***

To a solution of chromone-3-carb­oxy­lic acid (500 mg, 2.6 mmol) in DMF (4 ml) POCl_3_ (241 ml, 2.6 mmol) was added. The mixture was stirred at room temperature for 30 min, with the formation *in situ* of the corresponding acyl chloride. Then, the 5-methyl­thia­zol-2-amine was added. After 12 h, the mixture was diluted with di­chloro­methane (20 ml), washed with H_2_O (2 × 10 ml) and with saturated NaHCO_3_ solution (2 × 10 ml). The organic phase was dried with Na_2_SO_4_, filtered and concentrated under reduced pressure. The residue was purified by flash chromatography (20% ethyl acetate/petroleum ether) and *N*-(5-methyl­thia­zol-2-yl)-4-*oxo*-4*H*-chromene-3-carboxamide was obtained as a solid (153 mg, 20%). ^1^H NMR (400 MHz, CDCl_3_) δ 12.43 (NH, *s*, 1H), 9.05 (H3,*s*, 1H), 8.35 (H5, *dd*, *J* = 8.0, 1.5 Hz, 1H), 7.80 (H7, *ddd*, *J* = 8.7, 7.2, 1.5 Hz, 1H), 7.59 (H8, *dd*, *J* = 8.7, 1.6 Hz, 1H), 7.54 (H6, *ddd, J* = 8.0, 7.2, 1.0 Hz, 1H), 7.17 (H34, *q*, *J* = 1.2 Hz, 1H), 2.43 (CH_3_, *d*, *J* = 1.2 Hz, 3H). ^13^C NMR (101 MHz, CDCl_3_) δ 176.7 C4), 163.0 (C2), 160.3 (C31), 156.2 (C32), 155.5 (C8a), 135.4 (C7), 135.2 (C34), 128.4 (C35), 126.9 (C5), 126.7 (C6), 124.1 (C4a, C), 118.6 (C8), 114.6 (C3), 11.7 (CH_3_). EM/IE *m*/*z*: 287 (M^+^+1, 30), 286.0 (*M* ^+^, 91), 174 (30), 173 (100

## Refinement   

Crystal data, data collection and structure refinement details are summarized in Table 6 ▸. Crystals of the title compound with different morphologies were found in the crystallized sample. In each case several attempts were made at obtaining crystals which gave the best available data set for both types of morphology; **1_** ***P*** **2_1_/** ***n***: the crystals were long needles, which could not be cut, as they shattered. The needle used showed slight streaking on the images. The high angle data were very weak, with significant drop in intensity from the lower angle reflections. These facts probably explain the relatively high *R*-factor in the refinement of this compound. The following reflections were omitted from the refinement: 0 0 2 and 0 1 1 that were obstructed by beamstop and 0 10 1, 0 11 1, 0 12 1, 0 11 3 as recommend by the PLAT934_ALERT_3_B because (*I*_obs_ - *I*_calc_)/Σ *w* > 1.

**1_** ***P*** **2_1_/** ***c***: the crystals were prismatic in habit. The following reflections were omitted from the refinement: 0 1 1 obstructed by beamstop, 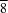 1 8, 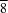 0 6 as recommend by the PLAT934_ALERT_3_B because (*I*_obs_ - *I*_calc_)/Σ *w* > 10.

The hydrogen atoms attached to the carboxamide N atom in **1_** ***P*** **2_1_/** ***n*** were treated as riding atoms with N—H = 0.88 Å and *U*_iso_(H) = 1.2*U*_eq_(N) while those in **1_** ***P*** **2_1_/** ***c*** were refined. All other H atoms were treated as riding atoms with C—H(aromatic) = 0.95 Å C—H(meth­yl) = 0.98 Å with *U*_iso_(H) = 1.5*U*_eq_(C). The positions of the amino and methyl hydrogen-atom positions were checked on a final difference map.

## Supplementary Material

Crystal structure: contains datablock(s) 1_P2~1~_n, 1_P2~1~_c, global. DOI: 10.1107/S2056989017009902/hb7689sup1.cif

Structure factors: contains datablock(s) 1_P2~1~_n. DOI: 10.1107/S2056989017009902/hb76891_P21_nsup2.hkl

Structure factors: contains datablock(s) 1_P2~1~_c. DOI: 10.1107/S2056989017009902/hb76891_P21_csup3.hkl

Gaseous phase quantum chemical calculations. DOI: 10.1107/S2056989017009902/hb7689sup4.pdf

CCDC references: 1560084, 1517021

Additional supporting information:  crystallographic information; 3D view; checkCIF report

## Figures and Tables

**Table 1:** Hydrogen-bond geometry (Å, °) for *P*2_1_/*n* [Chem scheme1]

*D*—H⋯*A*	*D*—H	H⋯*A*	*D*⋯*A*	*D*—H⋯*A*
N3—H3⋯O4	0.88	1.96	2.687 (5)	139
C2—H2⋯O4^i^	0.95	2.38	3.030 (5)	126
C8—H8⋯N33^i^	0.95	2.56	3.455 (6)	157

Symmetry code: (i) 
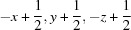
.

**Table 2:** Hydrogen-bond geometry (Å, °) for 1_P2~1~_c[Chem scheme1]

*D*—H⋯*A*	*D*—H	H⋯*A*	*D*⋯*A*	*D*—H⋯*A*
N13—H13⋯O14	0.80 (4)	1.99 (4)	2.671 (3)	143 (4)
C12—H12⋯O24^i^	0.95	2.27	2.963 (4)	129
C15—H15⋯O23^ii^	0.95	2.43	3.353 (4)	164
C136—H13*C*⋯O13^iii^	0.98	2.51	3.449 (4)	162
N23—H23⋯O24	0.82 (4)	2.05 (3)	2.697 (3)	136 (3)
C22—H22⋯O14^ii^	0.95	2.19	2.999 (4)	142
C25—H25⋯O13^iv^	0.95	2.48	3.352 (4)	153

Symmetry codes: (i) 
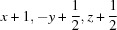
; (ii) 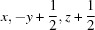; (iii) 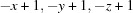; (iv) 
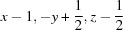
.

**Table 3:** Dihedral angles (°) θ_A–C_ is the dihedral angle between the mean planes of the chromene and phenyl ring and the thia­zole ring. θ_A–B_ is the dihedral angles between the mean planes of the chromone ring and the plane defined by the O2/C21/N2 atoms. θ_B–C_ is the dihedral angle between the mean planes of the thia­zole ring and the plane defined by the O3/C41/N3 atoms.

Compound	θ_A–C_°	θ_A–B_°	θ_B–C_°
**1_*P*2_1_/*n***	3.1 (2)	1.6 (8)	4.5 (8)
**1_*P*2_1_/*c*(mol#1)**	6.38 (9)	5.12 (19)	1.76 (12)
**1_*P*2_1_/*c*(mol#2)**	3.42 (10)	1.43 (10)	2.01 (14)

**Table 4:** Selected π–π contacts (Å) *CgI*(*J*) = Plane number *I*(*J*), *CgI*_Perp = perpendicular distance of *Cg*(*I*) on ring *J*, *CgJ*_Perp = perpendicular distance of *Cg*(*J*) on ring *I*, slippage = distance between *Cg*(*I*) and perpendicular projection of *Cg*(*J*) on Ring *I*.

Compound	*CgI*	*CgJ*(aru)	*Cg*⋯*Cg*	*CgI*_Perp	*CgJ*_Perp	Slippage
1_*P*2_1_/*n*	*Cg*1	*Cg*2(*x* + 1, *y*, *z*)	3.547 (3)	3.3358 (18)	3.3299 (19)	1.222
1_*P*2_1_/*c*	*Cg*3	*Cg*4(*x*, *y*, *z*)	3.6726 (17)	3.2645 (13)	3.3012 (12)	1.609

*Cg*1 and *Cg*2 are the centroids of rings O1/C2–C4/C4*A*/C8*A* and C4*A*/C5–C8/C8*A*, respectively. *Cg*3 and *Cg*4 are the centroids of rings C14*A*/C15–C18/C18*A* and O21/C22–C24/C24*A*/C28*A*, respectively.

**Table 5:** Percentages for the most relevant atom–atom contacts for the studied compounds

Compound	H⋯H	H⋯O/O⋯H	H⋯N/N⋯H	H⋯S/S⋯H	H⋯C/C⋯H	C⋯C
**1_*P*2_1_/*n***	31.8	19.0	8.7	6.3	15.2	7.1
**1_*P*2_1_/*c***	29.6	19.1	8.6	7.7	17.6	5.2
**1_*P*2_1_/*c***	34.6	13.2	6.6	7.1	18.4	5.2

**Table 6:** Experimental details

	*P*2_1_/*n*	*P*2_1_/*c*
Crystal data		
Chemical formula	C_14_H_10_N_2_O_3_S	C_14_H_10_N_2_O_3_S
*M*_r_	286.30	286.30
Crystal system, space group	Monoclinic, *P*2_1_/*n*	Monoclinic, *P*2_1_/*c*
Temperature (K)	100	100
*a*, *b*, *c* (Å)	4.8722 (4), 12.0436 (10), 21.9803 (16)	7.4646 (5), 30.626 (2), 11.0869 (8)
β (°)	96.353 (8)	93.232 (2)
*V* (Å^3^)	1281.86 (18)	2530.6 (3)
*Z*	4	8
Radiation type	Mo *K*α	Mo *K*α
μ (mm^−1^)	0.26	0.26
Crystal size (mm)	0.28 × 0.03 × 0.02	0.06 × 0.06 × 0.01

Data collection		
Diffractometer	Rigaku Saturn724+	Rigaku Saturn724+
Absorption correction	Multi-scan (*CrystalClear-SM Expert*; Rigaku, 2012 ▸)	Multi-scan (*CrystalClear-SM Expert*; Rigaku, 2012 ▸)
*T*_min_, *T*_max_	0.814, 1.000	0.538, 1.000
No. of measured, independent and observed [*I* > 2σ(*I*)] reflections	13600, 2944, 1980	15726, 5716, 3733
*R*_int_	0.084	0.090
(sin θ/λ)_max_ (Å^−1^)	0.651	0.650

Refinement		
*R*[*F* ^2^ > 2σ(*F* ^2^)], *wR*(*F* ^2^), *S*	0.097, 0.223, 1.13	0.060, 0.153, 1.08
No. of reflections	2944	5716
No. of parameters	182	371
H-atom treatment	H-atom parameters constrained	H atoms treated by a mixture of independent and constrained refinement
Δρ_max_, Δρ_min_ (e Å^−3^)	1.15, −0.37	0.46, −0.37

Computer programs: *CrystalClear-SM Expert* (Rigaku, 2012 ▸), *SHELXT* (Sheldrick, 2015*a* ▸), *SHELXL2014* (Sheldrick, 2015*b* ▸), *SHELXS* and *SHELXL* (Sheldrick, 2008 ▸), *ShelXle* (Hübschle *et al.*, 2011 ▸), *OSCAIL* (McArdle *et al.*, 2004 ▸), *Mercury* (Macrae *et al.*, 2006 ▸) and *PLATON* (Spek, 2009 ▸).

## supplementary crystallographic information

### N-(5-Methylthiazol-2-yl)-4-oxo-4H-chromene-3-carboxamide (1_P2~1~_n) Crystal data

**Table:**

C_14_H_10_N_2_O_3_S	*F*(000) = 592
*M**_r_* = 286.30	*D*_x_ = 1.484 Mg m^−^^3^
Monoclinic, *P*2_1_/*n*	Mo *K*α radiation, λ = 0.71073 Å
*a* = 4.8722 (4) Å	Cell parameters from 3369 reflections
*b* = 12.0436 (10) Å	θ = 2.5–27.5°
*c* = 21.9803 (16) Å	µ = 0.26 mm^−^^1^
β = 96.353 (8)°	*T* = 100 K
*V* = 1281.86 (18) Å^3^	Needle, yellow
*Z* = 4	0.28 × 0.03 × 0.02 mm

### N-(5-Methylthiazol-2-yl)-4-oxo-4H-chromene-3-carboxamide (1_P2~1~_n) Data collection

**Table:**

Rigaku Saturn724+ diffractometer	2944 independent reflections
Radiation source: Enhance (Mo) X-ray Source	1980 reflections with *I* > 2σ(*I*)
Graphite monochromator	*R*_int_ = 0.084
profile data from ω–scans	θ_max_ = 27.5°, θ_min_ = 2.5°
Absorption correction: multi-scan (*CrystalClear-SM Expert*; Rigaku, 2012)	*h* = −6→6
*T*_min_ = 0.814, *T*_max_ = 1.000	*k* = −15→15
13600 measured reflections	*l* = −28→28

### N-(5-Methylthiazol-2-yl)-4-oxo-4H-chromene-3-carboxamide (1_P2~1~_n) Refinement

**Table:**

Refinement on *F*^2^	0 restraints
Least-squares matrix: full	Hydrogen site location: inferred from neighbouring sites
*R*[*F*^2^ > 2σ(*F*^2^)] = 0.097	H-atom parameters constrained
*wR*(*F*^2^) = 0.223	*w* = 1/[σ^2^(*F*_o_^2^) + (0.0714*P*)^2^ + 3.9146*P*] where *P* = (*F*_o_^2^ + 2*F*_c_^2^)/3
*S* = 1.13	(Δ/σ)_max_ = 0.001
2944 reflections	Δρ_max_ = 1.15 e Å^−^^3^
182 parameters	Δρ_min_ = −0.37 e Å^−^^3^

### N-(5-Methylthiazol-2-yl)-4-oxo-4H-chromene-3-carboxamide (1_P2~1~_n) Special details

**Table:**

Geometry. All esds (except the esd in the dihedral angle between two l.s. planes) are estimated using the full covariance matrix. The cell esds are taken into account individually in the estimation of esds in distances, angles and torsion angles; correlations between esds in cell parameters are only used when they are defined by crystal symmetry. An approximate (isotropic) treatment of cell esds is used for estimating esds involving l.s. planes.

### N-(5-Methylthiazol-2-yl)-4-oxo-4H-chromene-3-carboxamide (1_P2~1~_n) Fractional atomic coordinates and isotropic or equivalent isotropic displacement parameters (Å^2^)

**Table:**

	*x*	*y*	*z*	*U*_iso_*/*U*_eq_	
S31	0.9829 (3)	0.89007 (11)	0.43688 (5)	0.0282 (3)	
O1	−0.0148 (6)	1.0107 (2)	0.21301 (14)	0.0251 (7)	
O3	0.6328 (7)	0.9809 (3)	0.34453 (14)	0.0297 (8)	
O4	0.1136 (6)	0.7088 (3)	0.29518 (14)	0.0252 (7)	
N3	0.5557 (8)	0.7992 (3)	0.36084 (16)	0.0245 (9)	
H3	0.4531	0.7413	0.3489	0.029*	
N33	0.8145 (9)	0.6867 (4)	0.43227 (18)	0.0323 (10)	
C2	0.1942 (10)	0.9927 (4)	0.2566 (2)	0.0245 (10)	
H2	0.3134	1.0535	0.2678	0.029*	
C3	0.2503 (9)	0.8961 (4)	0.28596 (19)	0.0221 (10)	
C4	0.0784 (9)	0.7999 (4)	0.27017 (19)	0.0211 (9)	
C5	−0.3234 (10)	0.7340 (4)	0.1989 (2)	0.0253 (10)	
H5	−0.2989	0.6614	0.2154	0.030*	
C4A	−0.1452 (9)	0.8189 (4)	0.22095 (19)	0.0213 (10)	
C6	−0.5338 (10)	0.7543 (4)	0.1535 (2)	0.0274 (11)	
H6	−0.6547	0.6959	0.1389	0.033*	
C7	−0.5705 (10)	0.8606 (4)	0.1288 (2)	0.0272 (11)	
H7	−0.7176	0.8742	0.0976	0.033*	
C8	−0.3976 (10)	0.9455 (4)	0.1489 (2)	0.0264 (10)	
H8	−0.4214	1.0179	0.1320	0.032*	
C8A	−0.1864 (10)	0.9224 (4)	0.1947 (2)	0.0237 (10)	
C31	0.4964 (9)	0.8976 (4)	0.33283 (19)	0.0224 (10)	
C32	0.7694 (10)	0.7843 (4)	0.4073 (2)	0.0283 (11)	
C34	1.0346 (11)	0.6953 (5)	0.4779 (2)	0.0357 (13)	
H34	1.1015	0.6326	0.5012	0.043*	
C35	1.1488 (10)	0.7959 (4)	0.4877 (2)	0.0304 (11)	
C351	1.3848 (10)	0.8291 (5)	0.5342 (2)	0.0368 (13)	
H35A	1.4522	0.7638	0.5579	0.055*	
H35B	1.3213	0.8851	0.5618	0.055*	
H35C	1.5347	0.8601	0.5131	0.055*	

### N-(5-Methylthiazol-2-yl)-4-oxo-4H-chromene-3-carboxamide (1_P2~1~_n) Atomic displacement parameters (Å^2^)

**Table:**

	*U*^11^	*U*^22^	*U*^33^	*U*^12^	*U*^13^	*U*^23^
S31	0.0245 (7)	0.0323 (7)	0.0281 (6)	0.0009 (6)	0.0043 (5)	−0.0015 (5)
O1	0.0252 (19)	0.0153 (16)	0.0340 (17)	−0.0006 (14)	−0.0002 (14)	0.0028 (13)
O3	0.030 (2)	0.0216 (18)	0.0369 (18)	−0.0055 (15)	0.0001 (14)	−0.0001 (14)
O4	0.0245 (19)	0.0164 (16)	0.0349 (17)	0.0041 (14)	0.0040 (14)	0.0008 (13)
N3	0.024 (2)	0.020 (2)	0.0289 (19)	0.0002 (17)	0.0021 (16)	−0.0030 (15)
N33	0.035 (3)	0.029 (2)	0.032 (2)	0.006 (2)	−0.0008 (18)	−0.0040 (17)
C2	0.024 (3)	0.017 (2)	0.034 (2)	−0.002 (2)	0.007 (2)	−0.0012 (18)
C3	0.023 (3)	0.017 (2)	0.028 (2)	0.0020 (19)	0.0098 (18)	0.0005 (17)
C4	0.022 (2)	0.015 (2)	0.028 (2)	0.0033 (19)	0.0093 (18)	−0.0021 (17)
C5	0.026 (3)	0.020 (2)	0.031 (2)	0.001 (2)	0.009 (2)	0.0017 (18)
C4A	0.023 (3)	0.015 (2)	0.028 (2)	−0.0045 (19)	0.0098 (19)	−0.0027 (17)
C6	0.025 (3)	0.025 (3)	0.033 (2)	−0.003 (2)	0.005 (2)	−0.0058 (19)
C7	0.027 (3)	0.027 (3)	0.028 (2)	0.001 (2)	0.003 (2)	0.0012 (18)
C8	0.026 (3)	0.021 (2)	0.033 (2)	0.005 (2)	0.004 (2)	0.0036 (18)
C8A	0.024 (3)	0.017 (2)	0.032 (2)	−0.0016 (19)	0.010 (2)	−0.0032 (18)
C31	0.023 (3)	0.017 (2)	0.028 (2)	0.000 (2)	0.0077 (19)	−0.0023 (17)
C32	0.033 (3)	0.026 (3)	0.027 (2)	0.004 (2)	0.008 (2)	−0.0043 (18)
C34	0.037 (3)	0.042 (3)	0.026 (2)	0.020 (3)	0.000 (2)	−0.002 (2)
C35	0.025 (3)	0.039 (3)	0.028 (2)	0.007 (2)	0.006 (2)	−0.004 (2)
C351	0.027 (3)	0.048 (3)	0.034 (3)	0.004 (3)	0.001 (2)	−0.005 (2)

### N-(5-Methylthiazol-2-yl)-4-oxo-4H-chromene-3-carboxamide (1_P2~1~_n) Geometric parameters (Å, º)

**Table:**

S31—C32	1.726 (5)	C5—C6	1.371 (7)
S31—C35	1.728 (5)	C5—C4A	1.393 (6)
O1—C2	1.336 (6)	C5—H5	0.9500
O1—C8A	1.384 (5)	C4A—C8A	1.379 (6)
O3—C31	1.215 (5)	C6—C7	1.394 (7)
O4—C4	1.231 (5)	C6—H6	0.9500
N3—C31	1.353 (6)	C7—C8	1.367 (7)
N3—C32	1.387 (6)	C7—H7	0.9500
N3—H3	0.8800	C8—C8A	1.387 (7)
N33—C32	1.306 (6)	C8—H8	0.9500
N33—C34	1.388 (6)	C34—C35	1.341 (7)
C2—C3	1.344 (6)	C34—H34	0.9500
C2—H2	0.9500	C35—C351	1.506 (7)
C3—C4	1.449 (6)	C351—H35A	0.9800
C3—C31	1.492 (7)	C351—H35B	0.9800
C4—C4A	1.466 (6)	C351—H35C	0.9800
			
C32—S31—C35	88.8 (2)	C6—C7—H7	119.5
C2—O1—C8A	118.1 (4)	C7—C8—C8A	117.8 (4)
C31—N3—C32	123.6 (4)	C7—C8—H8	121.1
C31—N3—H3	118.2	C8A—C8—H8	121.1
C32—N3—H3	118.2	C4A—C8A—O1	121.2 (4)
C32—N33—C34	108.4 (4)	C4A—C8A—C8	123.2 (4)
O1—C2—C3	125.7 (4)	O1—C8A—C8	115.6 (4)
O1—C2—H2	117.2	O3—C31—N3	122.9 (4)
C3—C2—H2	117.2	O3—C31—C3	122.4 (4)
C2—C3—C4	119.7 (4)	N3—C31—C3	114.8 (4)
C2—C3—C31	115.2 (4)	N33—C32—N3	120.2 (4)
C4—C3—C31	125.0 (4)	N33—C32—S31	116.1 (4)
O4—C4—C3	123.9 (4)	N3—C32—S31	123.7 (4)
O4—C4—C4A	121.6 (4)	C35—C34—N33	117.2 (5)
C3—C4—C4A	114.5 (4)	C35—C34—H34	121.4
C6—C5—C4A	120.7 (4)	N33—C34—H34	121.4
C6—C5—H5	119.7	C34—C35—C351	128.4 (5)
C4A—C5—H5	119.7	C34—C35—S31	109.4 (4)
C8A—C4A—C5	117.5 (4)	C351—C35—S31	122.1 (4)
C8A—C4A—C4	120.7 (4)	C35—C351—H35A	109.5
C5—C4A—C4	121.8 (4)	C35—C351—H35B	109.5
C5—C6—C7	120.0 (5)	H35A—C351—H35B	109.5
C5—C6—H6	120.0	C35—C351—H35C	109.5
C7—C6—H6	120.0	H35A—C351—H35C	109.5
C8—C7—C6	120.9 (5)	H35B—C351—H35C	109.5
C8—C7—H7	119.5		
			
C8A—O1—C2—C3	−1.8 (6)	C2—O1—C8A—C8	−179.3 (4)
O1—C2—C3—C4	0.9 (7)	C7—C8—C8A—C4A	−0.5 (7)
O1—C2—C3—C31	179.9 (4)	C7—C8—C8A—O1	179.3 (4)
C2—C3—C4—O4	−179.2 (4)	C32—N3—C31—O3	2.9 (7)
C31—C3—C4—O4	1.9 (7)	C32—N3—C31—C3	−177.4 (4)
C2—C3—C4—C4A	1.1 (6)	C2—C3—C31—O3	1.5 (6)
C31—C3—C4—C4A	−177.8 (4)	C4—C3—C31—O3	−179.6 (4)
C6—C5—C4A—C8A	−1.1 (6)	C2—C3—C31—N3	−178.2 (4)
C6—C5—C4A—C4	178.9 (4)	C4—C3—C31—N3	0.7 (6)
O4—C4—C4A—C8A	178.1 (4)	C34—N33—C32—N3	−178.9 (4)
C3—C4—C4A—C8A	−2.2 (6)	C34—N33—C32—S31	−0.4 (5)
O4—C4—C4A—C5	−1.9 (6)	C31—N3—C32—N33	180.0 (4)
C3—C4—C4A—C5	177.8 (4)	C31—N3—C32—S31	1.6 (6)
C4A—C5—C6—C7	0.2 (7)	C35—S31—C32—N33	−0.2 (4)
C5—C6—C7—C8	0.5 (7)	C35—S31—C32—N3	178.3 (4)
C6—C7—C8—C8A	−0.4 (7)	C32—N33—C34—C35	0.9 (6)
C5—C4A—C8A—O1	−178.5 (4)	N33—C34—C35—C351	179.2 (4)
C4—C4A—C8A—O1	1.5 (6)	N33—C34—C35—S31	−1.1 (6)
C5—C4A—C8A—C8	1.2 (7)	C32—S31—C35—C34	0.7 (4)
C4—C4A—C8A—C8	−178.8 (4)	C32—S31—C35—C351	−179.5 (4)
C2—O1—C8A—C4A	0.5 (6)		

### N-(5-Methylthiazol-2-yl)-4-oxo-4H-chromene-3-carboxamide (1_P2~1~_n) Hydrogen-bond geometry (Å, º)

**Table:**

*D*—H···*A*	*D*—H	H···*A*	*D*···*A*	*D*—H···*A*
N3—H3···O4	0.88	1.96	2.687 (5)	139
C2—H2···O4^i^	0.95	2.38	3.030 (5)	126
C8—H8···N33^i^	0.95	2.56	3.455 (6)	157

Symmetry code: (i) −*x*+1/2, *y*+1/2, −*z*+1/2.

### (1_P2~1~_c) Crystal data

**Table:**

C_14_H_10_N_2_O_3_S	*F*(000) = 1184
*M**_r_* = 286.30	*D*_x_ = 1.503 Mg m^−^^3^
Monoclinic, *P*2_1_/*c*	Mo *K*α radiation, λ = 0.71075 Å
*a* = 7.4646 (5) Å	Cell parameters from 12722 reflections
*b* = 30.626 (2) Å	θ = 2.3–27.5°
*c* = 11.0869 (8) Å	µ = 0.26 mm^−^^1^
β = 93.232 (2)°	*T* = 100 K
*V* = 2530.6 (3) Å^3^	Plate, colourless
*Z* = 8	0.06 × 0.06 × 0.01 mm

### (1_P2~1~_c) Data collection

**Table:**

Rigaku Saturn724+ diffractometer	5716 independent reflections
Radiation source: Sealed Tube	3733 reflections with *I* > 2σ(*I*)
Graphite Monochromator monochromator	*R*_int_ = 0.090
Detector resolution: 28.5714 pixels mm^-1^	θ_max_ = 27.5°, θ_min_ = 2.3°
profile data from ω–scans	*h* = −8→9
Absorption correction: multi-scan (*CrystalClear-SM Expert*; Rigaku, 2012)	*k* = −37→39
*T*_min_ = 0.538, *T*_max_ = 1.000	*l* = −14→14
15726 measured reflections	

### (1_P2~1~_c) Refinement

**Table:**

Refinement on *F*^2^	0 restraints
Least-squares matrix: full	Hydrogen site location: mixed
*R*[*F*^2^ > 2σ(*F*^2^)] = 0.060	H atoms treated by a mixture of independent and constrained refinement
*wR*(*F*^2^) = 0.153	*w* = 1/[σ^2^(*F*_o_^2^) + (0.0558*P*)^2^ + 1.3609*P*] where *P* = (*F*_o_^2^ + 2*F*_c_^2^)/3
*S* = 1.08	(Δ/σ)_max_ < 0.001
5716 reflections	Δρ_max_ = 0.46 e Å^−^^3^
371 parameters	Δρ_min_ = −0.37 e Å^−^^3^

### (1_P2~1~_c) Special details

**Table:**

Geometry. All esds (except the esd in the dihedral angle between two l.s. planes) are estimated using the full covariance matrix. The cell esds are taken into account individually in the estimation of esds in distances, angles and torsion angles; correlations between esds in cell parameters are only used when they are defined by crystal symmetry. An approximate (isotropic) treatment of cell esds is used for estimating esds involving l.s. planes.

### (1_P2~1~_c) Fractional atomic coordinates and isotropic or equivalent isotropic displacement parameters (Å^2^)

**Table:**

	*x*	*y*	*z*	*U*_iso_*/*U*_eq_	
S131	0.61048 (10)	0.46741 (2)	0.38319 (7)	0.01951 (19)	
S231	0.12461 (10)	0.35373 (2)	0.43837 (8)	0.0231 (2)	
O11	0.7012 (3)	0.25605 (6)	0.53314 (18)	0.0193 (5)	
O14	0.4476 (3)	0.30431 (6)	0.22432 (19)	0.0210 (5)	
O21	0.1940 (3)	0.13877 (6)	0.52234 (19)	0.0211 (5)	
O24	−0.0676 (3)	0.19810 (6)	0.2322 (2)	0.0221 (5)	
O131	0.6665 (3)	0.38847 (6)	0.49383 (19)	0.0213 (5)	
O231	0.1912 (3)	0.27146 (7)	0.5128 (2)	0.0254 (5)	
N13	0.5367 (3)	0.38401 (8)	0.3050 (2)	0.0187 (6)	
N23	0.0312 (3)	0.27500 (8)	0.3342 (3)	0.0198 (6)	
N133	0.4688 (4)	0.44311 (8)	0.1763 (2)	0.0259 (6)	
N233	−0.0482 (3)	0.33985 (8)	0.2334 (2)	0.0232 (6)	
C12	0.6872 (4)	0.29895 (9)	0.5127 (3)	0.0177 (6)	
H12	0.7403	0.3180	0.5722	0.021*	
C13	0.6033 (4)	0.31749 (9)	0.4141 (3)	0.0166 (6)	
C14	0.5230 (4)	0.29002 (9)	0.3192 (3)	0.0161 (6)	
C15	0.4645 (4)	0.21215 (9)	0.2591 (3)	0.0182 (6)	
H15	0.4032	0.2216	0.1863	0.022*	
C14A	0.5380 (4)	0.24282 (9)	0.3424 (3)	0.0170 (6)	
C16	0.4824 (4)	0.16821 (9)	0.2840 (3)	0.0191 (7)	
H16	0.4344	0.1474	0.2275	0.023*	
C17	0.5706 (4)	0.15392 (9)	0.3917 (3)	0.0209 (7)	
H17	0.5808	0.1235	0.4078	0.025*	
C18	0.6425 (4)	0.18345 (9)	0.4745 (3)	0.0190 (6)	
H18	0.7022	0.1739	0.5477	0.023*	
C18A	0.6252 (4)	0.22810 (9)	0.4478 (3)	0.0163 (6)	
C22	0.1841 (4)	0.18235 (9)	0.5132 (3)	0.0204 (7)	
H22	0.2399	0.1992	0.5768	0.024*	
C23	0.1006 (4)	0.20402 (9)	0.4203 (3)	0.0171 (6)	
C24	0.0102 (4)	0.18040 (10)	0.3211 (3)	0.0183 (6)	
C25	−0.0718 (4)	0.10459 (9)	0.2489 (3)	0.0198 (6)	
H25	−0.1343	0.1165	0.1795	0.024*	
C24A	0.0162 (4)	0.13245 (9)	0.3338 (3)	0.0185 (6)	
C26	−0.0676 (4)	0.06024 (10)	0.2662 (3)	0.0245 (7)	
H26	−0.1286	0.0416	0.2090	0.029*	
C27	0.0260 (4)	0.04218 (10)	0.3673 (3)	0.0268 (7)	
H27	0.0280	0.0114	0.3784	0.032*	
C28	0.1153 (4)	0.06891 (10)	0.4511 (3)	0.0240 (7)	
H28	0.1811	0.0569	0.5190	0.029*	
C28A	0.1069 (4)	0.11384 (9)	0.4338 (3)	0.0192 (6)	
C132	0.5337 (4)	0.42830 (9)	0.2799 (3)	0.0189 (6)	
C134	0.4804 (4)	0.48808 (10)	0.1767 (3)	0.0277 (8)	
H134	0.4396	0.5048	0.1084	0.033*	
C135	0.5518 (4)	0.50732 (9)	0.2774 (3)	0.0210 (7)	
C136	0.5782 (4)	0.55514 (9)	0.3022 (3)	0.0264 (8)	
H13A	0.5545	0.5717	0.2273	0.040*	
H13B	0.7020	0.5603	0.3330	0.040*	
H13C	0.4953	0.5646	0.3624	0.040*	
C137	0.6058 (4)	0.36610 (9)	0.4097 (3)	0.0164 (6)	
C232	0.0293 (4)	0.32010 (9)	0.3258 (3)	0.0190 (6)	
C234	−0.0323 (4)	0.38469 (10)	0.2497 (3)	0.0255 (7)	
H234	−0.0807	0.4047	0.1910	0.031*	
C235	0.0546 (4)	0.39855 (10)	0.3522 (3)	0.0249 (7)	
C236	0.0899 (4)	0.44428 (10)	0.3955 (3)	0.0302 (8)	
H23A	0.0396	0.4650	0.3353	0.045*	
H23B	0.2196	0.4490	0.4071	0.045*	
H23C	0.0337	0.4488	0.4723	0.045*	
C237	0.1123 (4)	0.25267 (10)	0.4275 (3)	0.0190 (6)	
H13	0.499 (5)	0.3673 (12)	0.255 (3)	0.036 (11)*	
H23	−0.012 (5)	0.2620 (11)	0.275 (3)	0.032 (11)*	

### (1_P2~1~_c) Atomic displacement parameters (Å^2^)

**Table:**

	*U*^11^	*U*^22^	*U*^33^	*U*^12^	*U*^13^	*U*^23^
S131	0.0231 (4)	0.0159 (3)	0.0195 (4)	0.0003 (3)	0.0008 (3)	−0.0002 (3)
S231	0.0199 (4)	0.0218 (4)	0.0277 (5)	0.0002 (3)	0.0006 (3)	−0.0048 (3)
O11	0.0246 (10)	0.0178 (10)	0.0152 (12)	−0.0001 (8)	−0.0014 (9)	0.0000 (9)
O14	0.0267 (11)	0.0184 (10)	0.0171 (12)	0.0018 (8)	−0.0062 (10)	0.0013 (9)
O21	0.0243 (11)	0.0237 (11)	0.0152 (12)	0.0017 (9)	−0.0007 (9)	0.0011 (9)
O24	0.0275 (11)	0.0217 (11)	0.0167 (12)	−0.0010 (9)	−0.0041 (10)	0.0007 (9)
O131	0.0270 (11)	0.0198 (10)	0.0167 (12)	−0.0008 (8)	−0.0016 (10)	−0.0025 (9)
O231	0.0274 (11)	0.0256 (11)	0.0224 (13)	0.0002 (9)	−0.0064 (10)	−0.0056 (10)
N13	0.0240 (14)	0.0158 (12)	0.0159 (14)	−0.0007 (10)	−0.0016 (12)	−0.0013 (11)
N23	0.0209 (13)	0.0196 (13)	0.0189 (15)	−0.0007 (10)	0.0010 (12)	−0.0030 (11)
N133	0.0344 (15)	0.0225 (13)	0.0202 (15)	0.0010 (11)	−0.0038 (13)	0.0032 (11)
N233	0.0266 (13)	0.0237 (13)	0.0197 (15)	0.0002 (11)	0.0040 (12)	0.0020 (11)
C12	0.0201 (14)	0.0166 (13)	0.0164 (16)	−0.0023 (11)	0.0006 (13)	−0.0027 (12)
C13	0.0156 (13)	0.0220 (14)	0.0125 (15)	−0.0005 (11)	0.0027 (12)	0.0000 (12)
C14	0.0154 (13)	0.0192 (14)	0.0138 (16)	0.0025 (11)	0.0015 (12)	−0.0011 (12)
C15	0.0179 (14)	0.0220 (14)	0.0149 (16)	−0.0012 (11)	0.0027 (13)	−0.0023 (12)
C14A	0.0153 (14)	0.0210 (14)	0.0151 (16)	0.0006 (11)	0.0053 (12)	−0.0003 (12)
C16	0.0210 (14)	0.0176 (14)	0.0191 (17)	−0.0033 (11)	0.0044 (13)	−0.0069 (12)
C17	0.0229 (15)	0.0171 (14)	0.0232 (18)	0.0006 (11)	0.0061 (14)	−0.0005 (13)
C18	0.0207 (14)	0.0199 (14)	0.0168 (16)	0.0004 (11)	0.0040 (13)	0.0032 (12)
C18A	0.0153 (13)	0.0241 (14)	0.0096 (15)	−0.0015 (11)	0.0011 (12)	−0.0043 (12)
C22	0.0193 (14)	0.0230 (15)	0.0189 (17)	−0.0013 (12)	0.0014 (13)	−0.0008 (13)
C23	0.0141 (13)	0.0235 (15)	0.0138 (16)	0.0002 (11)	0.0023 (12)	0.0005 (12)
C24	0.0170 (14)	0.0251 (15)	0.0128 (15)	−0.0011 (12)	0.0016 (12)	0.0020 (12)
C25	0.0190 (14)	0.0238 (14)	0.0163 (17)	−0.0013 (12)	−0.0013 (13)	−0.0008 (13)
C24A	0.0183 (14)	0.0230 (15)	0.0145 (16)	0.0020 (11)	0.0037 (13)	0.0009 (12)
C26	0.0276 (16)	0.0228 (15)	0.0231 (18)	−0.0022 (13)	0.0018 (15)	−0.0024 (14)
C27	0.0302 (17)	0.0193 (15)	0.031 (2)	0.0003 (13)	0.0061 (16)	0.0029 (14)
C28	0.0240 (15)	0.0268 (16)	0.0215 (18)	0.0046 (13)	0.0023 (14)	0.0058 (14)
C28A	0.0198 (14)	0.0219 (14)	0.0163 (16)	−0.0002 (11)	0.0043 (13)	−0.0002 (13)
C132	0.0188 (14)	0.0189 (14)	0.0190 (17)	−0.0004 (11)	0.0015 (13)	−0.0015 (13)
C134	0.0374 (18)	0.0185 (15)	0.027 (2)	0.0024 (13)	−0.0001 (16)	0.0053 (14)
C135	0.0196 (14)	0.0195 (14)	0.0242 (18)	0.0015 (12)	0.0047 (14)	0.0040 (13)
C136	0.0325 (17)	0.0174 (15)	0.030 (2)	−0.0011 (13)	0.0071 (16)	0.0035 (14)
C137	0.0128 (13)	0.0199 (14)	0.0162 (17)	−0.0013 (11)	−0.0011 (12)	−0.0006 (12)
C232	0.0187 (14)	0.0212 (14)	0.0172 (16)	−0.0003 (11)	0.0023 (13)	−0.0006 (13)
C234	0.0291 (17)	0.0229 (15)	0.0255 (19)	0.0014 (13)	0.0096 (15)	0.0009 (14)
C235	0.0200 (15)	0.0202 (15)	0.035 (2)	−0.0018 (12)	0.0093 (15)	−0.0016 (14)
C236	0.0242 (16)	0.0253 (16)	0.041 (2)	−0.0004 (13)	0.0024 (16)	−0.0040 (16)
C237	0.0158 (13)	0.0240 (15)	0.0172 (16)	0.0012 (11)	0.0010 (13)	−0.0035 (13)

### (1_P2~1~_c) Geometric parameters (Å, º)

**Table:**

S131—C132	1.732 (3)	C16—H16	0.9500
S131—C135	1.733 (3)	C17—C18	1.376 (4)
S231—C235	1.736 (3)	C17—H17	0.9500
S231—C232	1.739 (3)	C18—C18A	1.404 (4)
O11—C12	1.336 (3)	C18—H18	0.9500
O11—C18A	1.374 (3)	C22—C23	1.348 (4)
O14—C14	1.244 (4)	C22—H22	0.9500
O21—C22	1.340 (3)	C23—C24	1.450 (4)
O21—C28A	1.377 (4)	C23—C237	1.495 (4)
O24—C24	1.241 (4)	C24—C24A	1.476 (4)
O131—C137	1.223 (3)	C25—C26	1.372 (4)
O231—C237	1.229 (4)	C25—C24A	1.405 (4)
N13—C137	1.359 (4)	C25—H25	0.9500
N13—C132	1.385 (4)	C24A—C28A	1.389 (4)
N13—H13	0.80 (4)	C26—C27	1.401 (5)
N23—C237	1.354 (4)	C26—H26	0.9500
N23—C232	1.384 (4)	C27—C28	1.381 (4)
N23—H23	0.82 (4)	C27—H27	0.9500
N133—C132	1.302 (4)	C28—C28A	1.390 (4)
N133—C134	1.380 (4)	C28—H28	0.9500
N233—C232	1.297 (4)	C134—C135	1.345 (5)
N233—C234	1.389 (4)	C134—H134	0.9500
C12—C13	1.354 (4)	C135—C136	1.501 (4)
C12—H12	0.9500	C136—H13A	0.9800
C13—C14	1.451 (4)	C136—H13B	0.9800
C13—C137	1.490 (4)	C136—H13C	0.9800
C14—C14A	1.472 (4)	C234—C235	1.346 (5)
C15—C16	1.379 (4)	C234—H234	0.9500
C15—C14A	1.407 (4)	C235—C236	1.499 (4)
C15—H15	0.9500	C236—H23A	0.9800
C14A—C18A	1.381 (4)	C236—H23B	0.9800
C16—C17	1.401 (4)	C236—H23C	0.9800
			
C132—S131—C135	88.76 (15)	C28A—C24A—C25	118.3 (3)
C235—S231—C232	88.58 (16)	C28A—C24A—C24	119.7 (3)
C12—O11—C18A	118.0 (2)	C25—C24A—C24	122.0 (3)
C22—O21—C28A	118.4 (2)	C25—C26—C27	120.6 (3)
C137—N13—C132	124.6 (3)	C25—C26—H26	119.7
C137—N13—H13	116 (3)	C27—C26—H26	119.7
C132—N13—H13	119 (3)	C28—C27—C26	120.3 (3)
C237—N23—C232	123.9 (3)	C28—C27—H27	119.9
C237—N23—H23	120 (3)	C26—C27—H27	119.9
C232—N23—H23	116 (3)	C27—C28—C28A	118.6 (3)
C132—N133—C134	109.0 (3)	C27—C28—H28	120.7
C232—N233—C234	109.1 (3)	C28A—C28—H28	120.7
O11—C12—C13	125.3 (3)	O21—C28A—C24A	122.1 (3)
O11—C12—H12	117.3	O21—C28A—C28	115.8 (3)
C13—C12—H12	117.3	C24A—C28A—C28	122.1 (3)
C12—C13—C14	119.7 (3)	N133—C132—N13	121.3 (3)
C12—C13—C137	116.1 (3)	N133—C132—S131	115.8 (2)
C14—C13—C137	124.1 (3)	N13—C132—S131	122.9 (2)
O14—C14—C13	123.9 (3)	C135—C134—N133	117.5 (3)
O14—C14—C14A	121.3 (3)	C135—C134—H134	121.3
C13—C14—C14A	114.7 (3)	N133—C134—H134	121.3
C16—C15—C14A	119.3 (3)	C134—C135—C136	128.4 (3)
C16—C15—H15	120.3	C134—C135—S131	109.0 (2)
C14A—C15—H15	120.3	C136—C135—S131	122.6 (3)
C18A—C14A—C15	119.0 (3)	C135—C136—H13A	109.5
C18A—C14A—C14	119.8 (3)	C135—C136—H13B	109.5
C15—C14A—C14	121.2 (3)	H13A—C136—H13B	109.5
C15—C16—C17	120.8 (3)	C135—C136—H13C	109.5
C15—C16—H16	119.6	H13A—C136—H13C	109.5
C17—C16—H16	119.6	H13B—C136—H13C	109.5
C18—C17—C16	120.7 (3)	O131—C137—N13	122.1 (3)
C18—C17—H17	119.7	O131—C137—C13	122.6 (3)
C16—C17—H17	119.7	N13—C137—C13	115.2 (3)
C17—C18—C18A	118.1 (3)	N233—C232—N23	121.4 (3)
C17—C18—H18	120.9	N233—C232—S231	115.9 (2)
C18A—C18—H18	120.9	N23—C232—S231	122.7 (2)
O11—C18A—C14A	122.4 (3)	C235—C234—N233	117.1 (3)
O11—C18A—C18	115.6 (3)	C235—C234—H234	121.5
C14A—C18A—C18	122.0 (3)	N233—C234—H234	121.5
O21—C22—C23	124.7 (3)	C234—C235—C236	129.3 (3)
O21—C22—H22	117.7	C234—C235—S231	109.3 (2)
C23—C22—H22	117.7	C236—C235—S231	121.4 (3)
C22—C23—C24	120.6 (3)	C235—C236—H23A	109.5
C22—C23—C237	115.3 (3)	C235—C236—H23B	109.5
C24—C23—C237	124.1 (3)	H23A—C236—H23B	109.5
O24—C24—C23	124.2 (3)	C235—C236—H23C	109.5
O24—C24—C24A	121.4 (3)	H23A—C236—H23C	109.5
C23—C24—C24A	114.5 (3)	H23B—C236—H23C	109.5
C26—C25—C24A	120.1 (3)	O231—C237—N23	121.7 (3)
C26—C25—H25	119.9	O231—C237—C23	122.1 (3)
C24A—C25—H25	119.9	N23—C237—C23	116.1 (3)
			
C18A—O11—C12—C13	0.1 (4)	C22—O21—C28A—C28	−177.2 (2)
O11—C12—C13—C14	−1.3 (4)	C25—C24A—C28A—O21	−178.9 (2)
O11—C12—C13—C137	−179.4 (2)	C24—C24A—C28A—O21	−0.6 (4)
C12—C13—C14—O14	−178.1 (3)	C25—C24A—C28A—C28	1.0 (4)
C137—C13—C14—O14	−0.1 (4)	C24—C24A—C28A—C28	179.3 (2)
C12—C13—C14—C14A	1.5 (4)	C27—C28—C28A—O21	178.0 (2)
C137—C13—C14—C14A	179.4 (2)	C27—C28—C28A—C24A	−1.8 (4)
C16—C15—C14A—C18A	−0.5 (4)	C134—N133—C132—N13	179.2 (2)
C16—C15—C14A—C14	179.7 (2)	C134—N133—C132—S131	0.4 (3)
O14—C14—C14A—C18A	178.9 (2)	C137—N13—C132—N133	178.4 (3)
C13—C14—C14A—C18A	−0.6 (4)	C137—N13—C132—S131	−2.9 (4)
O14—C14—C14A—C15	−1.2 (4)	C135—S131—C132—N133	−0.5 (2)
C13—C14—C14A—C15	179.2 (2)	C135—S131—C132—N13	−179.2 (2)
C14A—C15—C16—C17	0.8 (4)	C132—N133—C134—C135	−0.1 (4)
C15—C16—C17—C18	−0.5 (4)	N133—C134—C135—C136	−179.3 (3)
C16—C17—C18—C18A	−0.1 (4)	N133—C134—C135—S131	−0.3 (4)
C12—O11—C18A—C14A	0.8 (4)	C132—S131—C135—C134	0.4 (2)
C12—O11—C18A—C18	−179.4 (2)	C132—S131—C135—C136	179.5 (2)
C15—C14A—C18A—O11	179.6 (2)	C132—N13—C137—O131	2.8 (4)
C14—C14A—C18A—O11	−0.5 (4)	C132—N13—C137—C13	−176.9 (2)
C15—C14A—C18A—C18	−0.1 (4)	C12—C13—C137—O131	−5.8 (4)
C14—C14A—C18A—C18	179.7 (2)	C14—C13—C137—O131	176.1 (3)
C17—C18—C18A—O11	−179.3 (2)	C12—C13—C137—N13	173.8 (2)
C17—C18—C18A—C14A	0.4 (4)	C14—C13—C137—N13	−4.2 (4)
C28A—O21—C22—C23	−2.9 (4)	C234—N233—C232—N23	179.0 (2)
O21—C22—C23—C24	1.0 (4)	C234—N233—C232—S231	0.5 (3)
O21—C22—C23—C237	−178.0 (2)	C237—N23—C232—N233	179.0 (3)
C22—C23—C24—O24	−179.2 (3)	C237—N23—C232—S231	−2.6 (4)
C237—C23—C24—O24	−0.3 (4)	C235—S231—C232—N233	−0.4 (2)
C22—C23—C24—C24A	1.2 (4)	C235—S231—C232—N23	−178.9 (2)
C237—C23—C24—C24A	−180.0 (2)	C232—N233—C234—C235	−0.3 (4)
C26—C25—C24A—C28A	0.3 (4)	N233—C234—C235—C236	−178.9 (3)
C26—C25—C24A—C24	−177.9 (3)	N233—C234—C235—S231	0.0 (3)
O24—C24—C24A—C28A	179.0 (3)	C232—S231—C235—C234	0.2 (2)
C23—C24—C24A—C28A	−1.3 (4)	C232—S231—C235—C236	179.2 (2)
O24—C24—C24A—C25	−2.8 (4)	C232—N23—C237—O231	0.0 (4)
C23—C24—C24A—C25	176.9 (2)	C232—N23—C237—C23	−179.7 (2)
C24A—C25—C26—C27	−0.8 (4)	C22—C23—C237—O231	−0.6 (4)
C25—C26—C27—C28	0.0 (4)	C24—C23—C237—O231	−179.5 (3)
C26—C27—C28—C28A	1.3 (4)	C22—C23—C237—N23	179.2 (2)
C22—O21—C28A—C24A	2.7 (4)	C24—C23—C237—N23	0.3 (4)

### (1_P2~1~_c) Hydrogen-bond geometry (Å, º)

**Table:**

*D*—H···*A*	*D*—H	H···*A*	*D*···*A*	*D*—H···*A*
N13—H13···O14	0.80 (4)	1.99 (4)	2.671 (3)	143 (4)
C12—H12···O24^i^	0.95	2.27	2.963 (4)	129
C15—H15···O23^ii^	0.95	2.43	3.353 (4)	164
C136—H13*C*···O13^iii^	0.98	2.51	3.449 (4)	162
N23—H23···O24	0.82 (4)	2.05 (3)	2.697 (3)	136 (3)
C22—H22···O14^ii^	0.95	2.19	2.999 (4)	142
C25—H25···O13^iv^	0.95	2.48	3.352 (4)	153

Symmetry codes: (i) *x*+1, −*y*+1/2, *z*+1/2; (ii) *x*, −*y*+1/2, *z*+1/2; (iii) −*x*+1, −*y*+1, −*z*+1; (iv) *x*−1, −*y*+1/2, *z*−1/2.
