# Supplementary material for: Polymorphism in the structure of N-(5-methyl­thia­zol-2-yl)-4-oxo-4H-chromene-3-carboxamide
Source: Acta Crystallogr E Crystallogr Commun. 2017 Jul 13;73(Pt 8):1154–61. doi: 10.1107/S2056989017009902 (PMC5598839; doi:10.1107/S2056989017009902)
Supplement: Supplementary file 4 [file e-73-01154-sup4.pdf]

Structural studies on 4-oxo-*N*-5-methyl-1,3-thiazol-2-yl-4*H*-chromone -3-carboxamide

Supplementary Information

Lígia R. Gomes,[a] John Nicolson Low,[b]* Fernando Cagide[c] and Fernanda Borges[c]

[a]UFP Energy, Environment and Health Research Unit (FP-ENAS), Universidade Fernando Pessoa, Rua Carlos da Maia, 296, P-4200-150 Porto, Portugal, [b]Department of Chemistry, University of Aberdeen, Meston Walk, Old Aberdeen, AB24 3UE, Scotland, and [c]CIQ/Departamento de Química e Bioquímica, Faculdade de Ciências, Universidade do Porto, 4169-007 Porto, Portugal

Correspondence email: lrebelogomes@gmail.com

Gaseous phase quantum chemical calculations

Single point geometric energies calculations for **1_P2$_1$/n** at crystal geometry and for of conformers obtained by rotation of the thiazole ring (C) around the C-C bond connecting the B and C fragments were performed using density functional theory. The quantum chemical calculations were performed at the B3LYP exchange correlation functional, which combines the hybrid exchange functional of Becke (Becke, 1997) with the gradient-correlation functional of Lee, (Lee *et al*. 1998) and the split-valence polarised 6-311+G(d, p) basis set (Hehre, 1986) level of theory. The GaussView 3.0 (DenningtonII, program was used to get visual animation. All theoretical calculations were performed with the GAUSSIAN 03 program package (Frisch *et al*., 2003) The bonding characteristics of the compounds studied were investigated using natural population (NPA) analysis of Reed and Weinhold(Reed & Weinhold, 1983, Reed et al., 1998). Figure SI1 gives the graphic representation of the HF energy (with ZPE correction) for the conformers obtained by the rotation above mentioned. The lowest energy calculated corresponds to a rotation angle of 0º, that is when the S atom of the thiazole ring is in a –cis position with respect to the carbonyl oxygen atom of the amide, while the maximum energy computed corresponds to a rotation angle of 180º, when it is in a *trans* position.

The values for the atomic natural total charges have been retrieved at same theory level. Relevant values are presented in figure SI2 at crystal conformation. The inspection of the files for the different geometries obtained from the rotations around the C-C bond connecting the thiazole and the amide moiety revealed

that the change of charges for the S atom was the most sensitive. The lowest energy conformer (at 0º of rotation) showed the highest positive charge for the atom, as depicted in graphic representation of Figure SI3.

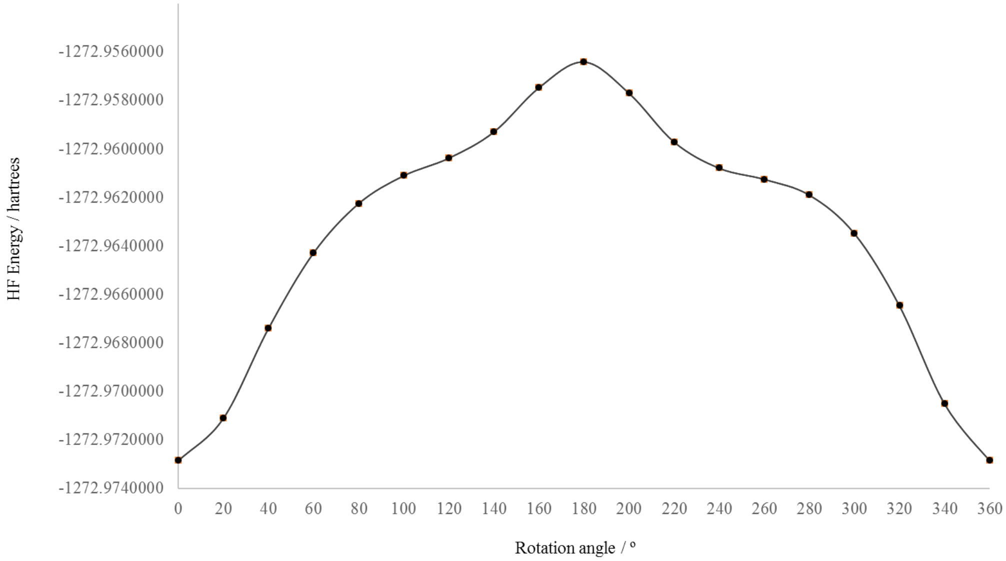
HF Energy / hartrees
-1272.9560000
-1272.9580000
-1272.9600000
-1272.9620000
-1272.9640000
-1272.9660000
-1272.9680000
-1272.9700000
-1272.9720000
-1272.9740000
0 20 40 60 80 100 120 140 160 180 200 220 240 260 280 300 320 340 360
Rotation angle / º

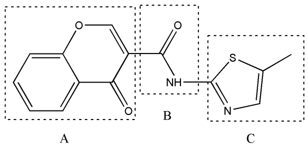
O
O
O
NH
S
N
A
B
C

Figure SI1 - HF energy at the B3LYP/6-311+G(d) level of theory of **1_P21/n** at conformations archived by rotation of the thiazole ring around the C-C bond connecting to the amide.

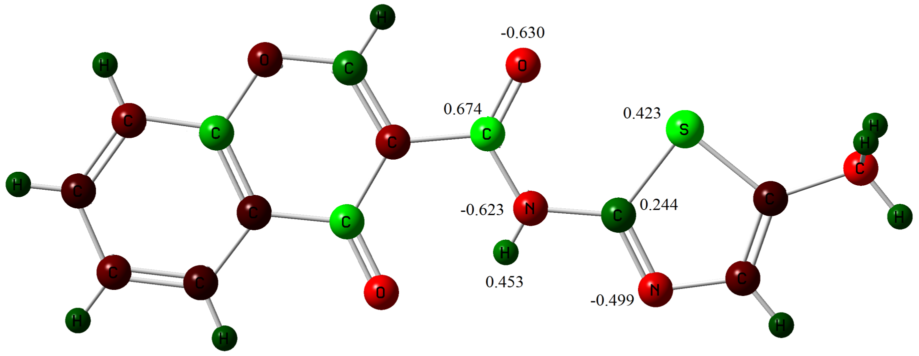

Figure SI2 - Natural atomic charges from population analysis (NBO), at the B3LYP/6-311+G(d) level of theory for **1_P21/n** at crystal geometric conformation. The charge distributions are presented within a relative charge range of -1.000 (green) to +1.000 (light red).

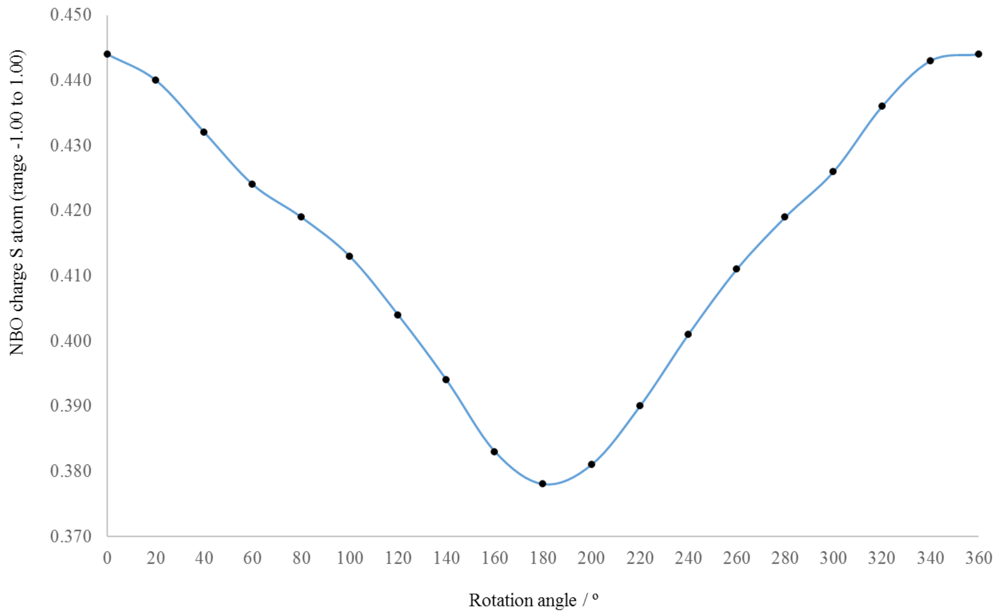
NBO charge S atom (range -1.00 to 1.00)
0.450
0.440
0.430
0.420
0.410
0.400
0.390
0.380
0.370
0
20
40
60
80
100
120
140
160
180
200
220
240
260
280
300
320
340
360
Rotation angle / º

Figure SI3 - Natural atomic charges from population analysis (NBO), at the B3LYP/6-311+G(d) level of theory for the S atom of **1_P21/n** at conformation archived by rotation of the thiazole ring around the C-C bond connecting to the amide. The charge distributions are presented within a relative charge range of -1.000 to +1.000.

Becke, A.D. (1997). *J Chem Phys* **107**, 8554--8560.

Lee, C., Yang , W. & Parr, W.G. (1998) *Phys Rev* **B37**, 785--789.

Hehre, W.J., Radom, L., Schleyer, P.V.R. & Pople, .J.A. (1986). Ab Initio Molecular Orbital Theory, Wiley, New York.

DenningtonII, R., Keith, T. & Millam, J. GaussView, Version 4.1.2, Semichem, Inc., Shawnee

Frisch, M.J., Trucks, G.W., Schlegel, H.B., Scuseria, G.E., Robb, M.A., Cheeseman, J.R., Montgomery, J.A., Vreven, T., Kudin, K.N., Burant, J.C., Millam, J.M., Iyengar,S.S., Tomasi, J., Barone, V., Mennucci, B., Cossi, M., Scalmani, G., Rega, N., Petersson, G.A., Nakatsuji, H., Hada, M., Ehara, M., Toyota, K., Fukuda, R., Hasegawa, J., Ishida, M., Nakajima,T., Honda, Y., Kitao, O., Nakai, H., Klene, M., Li, X., Knox, J.E., Hratchian, H.P., Cross, J.B., Adamo, C., Jaramillo, J., Gomperts, R., Stratmann, R.E., Yazyev, O., Austin, A.J., Cammi, R., Pomelli, C., Ochterski, W.J., Ayala, P.Y., Morokuma, K., Voth, G.A., Salvdor, P., Dannenberg, J.J., Zakrzewski, V.G., Dapprich, S., Daniels, A.D., Strain, M.C., Farkas, O., Malick, D.K., Rabuck, A.D., Raghavachari, K., Foresman, J.B., Ortiz, J.V., Cui, Q., Baboul, A.G., Clifford, S., Cioslowsk, J., Stefanov, B.B., Liu, G., Liashenko, A., Piskorz, P., Komaromi, I., Maring, R.L., Fox, D.J., Keith, T., Al-Laham, M.A., Peng, C.Y., Nanayakkara, A., Challacombe, M., Gill, P.M.W., Johnson, B., Chen, W., Wong, M.W., Gonzalez, C. & Pople, J.A. (2003). Gaussian 03, revision C1 [computer software]. Pittsburgh, PA, USA: Gaussian.

Reed, A.E. & Weinhold, F.J. (1983). *Chem. Phys*. **78**, 4066--4073.

Reed, A.E., Curtiss , L.A. & Weinhold, F. (1988) . *Chem. Rev*. **88,** 899--926.

Datablock: P21/n

```
Bond precision:      C-C = 0.0069 A              Wavelength=0.71075
Cell:          a=4.8722(4)     b=12.0436(10)  c=21.9803(16)
               alpha=90        beta=96.353(8) gamma=90
Temperature: 100 K
```

	Calculated	Reported
Volume	1281.86(18)	1281.86(18)
Space group	P 21/n	P 21/n
Hall group	-P 2yn	-P 2yn
Moiety formula	C14 H10 N2 O3 S	C14 H10 N2 O3 S
Sum formula	C14 H10 N2 O3 S	C14 H10 N2 O3 S
Mr	286.30	286.30
Dx,g cm-3	1.484	1.484
Z	4	4
Mu (mm-1)	0.261	0.261
F000	592.0	592.0
F000'	592.76	
h,k,lmax	6,15,28	6,15,28
Nref	2960	2948
Tmin,Tmax	0.991,0.995	0.814,1.000
Tmin'	0.930	

```
Correction method= # Reported T Limits: Tmin=0.814
Tmax=1.000 AbsCorr = MULTI-SCAN
Data completeness= 0.996        Theta(max)= 27.544
R(reflections)= 0.0977( 1984)      wR2(reflections)= 0.2251( 2945)
S = 1.141              Npar= 182
```

The following ALERTS were generated. Each ALERT has the format
test-name_ALERT_alert-type_alert-level.
Click on the hyperlinks for more details of the test.

Alert level C

PLAT094_ALERT_2_C Ratio of Maximum / Minimum Residual Density .... 3.24 Report
PLAT340_ALERT_3_C Low Bond Precision on C-C Bonds .... 0.00692 Ang.
PLAT906_ALERT_3_C Large K value in the Analysis of Variance ...... 24.783 Check
And 2 other PLAT906 Alerts
More ...
PLAT911_ALERT_3_C Missing # FCF Refl Between THmin & STh/L= 0.600 6 Report
PLAT934_ALERT_3_C Number of (Iobs-Icalc)/SigmaW > 10 Outliers .... 1 Check
PLAT978_ALERT_2_C Number C-C Bonds with Positive Residual Density. 0 Note

Alert level G

PLAT007_ALERT_5_G Number of Unrefined Donor-H Atoms .... 1 Report
PLAT910_ALERT_3_G Missing # of FCF Reflection(s) Below Theta(Min) 2 Note
PLAT912_ALERT_4_G Missing # of FCF Reflections Above STh/L= 0.600 8 Note

PLAT933_ALERT_2_G Number of OMIT Records in Embedded .res File ... 5
Note

0 **ALERT level A** = Most likely a serious problem - resolve or explain
0 **ALERT level B** = A potentially serious problem, consider carefully
8 **ALERT level C** = Check. Ensure it is not caused by an omission or oversight
4 **ALERT level G** = General information/check it is not something unexpected

0 ALERT type 1 CIF construction/syntax error, inconsistent or missing data
3 ALERT type 2 Indicator that the structure model may be wrong or deficient
7 ALERT type 3 Indicator that the structure quality may be low
1 ALERT type 4 Improvement, methodology, query or suggestion
1 ALERT type 5 Informative message, check

Datablock: P21/c

```
Bond precision:      C-C = 0.0042 A              Wavelength=0.71075
Cell:         a=7.4646(5)    b=30.626(2)    c=11.0869(8)
              alpha=90       beta=93.232(2) gamma=90
Temperature:100 K
                     Calculated                         Reported
Volume               2530.6(3)                          2530.6(3)
Space group          P 21/c                             P 21/c
Hall group           -P 2ybc                            -P 2ybc
Moiety formula       C14 H10 N2 O3 S                    C14 H10 N2 O3 S
Sum formula          C14 H10 N2 O3 S                    C14 H10 N2 O3 S
Mr                   286.30                             286.30
Dx,g cm-3            1.503                              1.503
Z                    8                                  8
Mu (mm-1)            0.264                              0.264
F000                 1184.0                             1184.0
F000'                1185.52
h,k,lmax             9,39,14                            9,39,14
Nref                 5834                               5716
Tmin,Tmax            0.984,0.997                        0.984,0.997
Tmin'                0.984
Correction method= # Reported T Limits: Tmin=0.984
Tmax=0.997 AbsCorr = MULTI-SCAN
Data completeness= 0.980        Theta(max)= 27.529
R(reflections)= 0.0598( 3733)      wR2(reflections)= 0.1532( 5716)
S = 1.078              Npar= 371
```

The following ALERTS were generated. Each ALERT has the format

test-name_ALERT_alert-type_alert-level.

Click on the hyperlinks for more details of the test.

Alert level C

PLAT340_ALERT_3_C Low Bond Precision on C-C Bonds .... 0.00421 Ang.

PLAT480_ALERT_4_C Long H...A H-Bond Reported H28 .. N133 .. 2.69 Ang.

PLAT480_ALERT_4_C Long H...A H-Bond Reported H23B .. S131 .. 3.00 Ang.

PLAT906_ALERT_3_C Large K value in the Analysis of Variance ...... 2.647 Check

PLAT911_ALERT_3_C Missing # FCF Refl Between THmin & STh/L= 0.600 49 Report

Alert level G

PLAT066_ALERT_1_G Predicted and Reported Tmin&Tmax Range Identical ? Check

PLAT432_ALERT_2_G Short Inter X...Y Contact O14 .. C22 .. 3.00 Ang.

PLAT432_ALERT_2_G Short Inter X...Y Contact O24 .. C12 .. 2.96 Ang.

PLAT910_ALERT_3_G Missing # of FCF Reflection(s) Below Theta(Min) 2 Note

PLAT912_ALERT_4_G Missing # of FCF Reflections Above STh/L= 0.600 67 Note

PLAT978_ALERT_2_G Number C-C Bonds with Positive Residual Density. 7 Note

0 **ALERT level A** = Most likely a serious problem - resolve or explain
0 **ALERT level B** = A potentially serious problem, consider carefully
5 **ALERT level C** = Check. Ensure it is not caused by an omission or oversight
6 **ALERT level G** = General information/check it is not something unexpected

1 ALERT type 1 CIF construction/syntax error, inconsistent or missing data
3 ALERT type 2 Indicator that the structure model may be wrong or deficient
4 ALERT type 3 Indicator that the structure quality may be low
3 ALERT type 4 Improvement, methodology, query or suggestion
0 ALERT type 5 Informative message, check
